# Expression characteristics of dual‐specificity phosphatase 2 and hypoxia‐inducible factor‐1α in acute kidney injury and preliminary study of the effect of dual‐specificity phosphatase 2 on HK‐2 cells

**DOI:** 10.1113/EP092493

**Published:** 2025-09-02

**Authors:** Xueqian Chu, Songmin Xue, Lanyan Lin, Xuan Huang, Sisi Chen, Wulaer Adeli, Zulibiya Tuohetiyaer, Suhua Li, Chen Lu

**Affiliations:** ^1^ Nephrology Disease Center, The First Affiliated Hospital of Xinjiang Medical University, State Key Laboratory of Pathogenesis Prevention and Treatment of High Incidence Diseases in Central Asia Urumqi Xinjiang China; ^2^ Xinjiang Clinical Research Center of Renal Replacement Therapy Urumqi Xinjiang China; ^3^ Xinjiang Branch of National Clinical Research Center for Kidney Disease Urumqi Xinjiang China; ^4^ Xinjiang Blood Purification Medical Quality Control Center Urumqi Xinjiang China; ^5^ Institute of Nephrology of Xinjiang Urumqi Xinjiang China; ^6^ Clinical Medicine (“5+3” Integration), Department of Clinical Medicine Xinjiang Medical University Urumqi Xinjiang China

**Keywords:** acute kidney injury, cell function, DUSP2, HIF‐1α

## Abstract

Acute kidney injury (AKI) is a global health problem with significant long‐term harm if the prognosis is poor. Dual‐specificity phosphatase 2 (DUSP2) is involved in key regulatory pathways in several disease processes, but its function in renal pathophysiology is unclear. The expression levels of DUSP2 in the peripheral blood of AKI and non‐AKI patients were first examined. The expression characteristics of DUSP2 and hypoxia‐inducible factor‐1α (HIF‐1α) in AKI conditions were analysed in renal tissues from AKI patients and different AKI model mice. Human renal tubular epithelial cells (HK‐2) were utilized for in vitro experiments to investigate the effects of overexpression of DUSP2 on cell proliferation, apoptosis and HIF‐1α levels under hypoxia–reoxygenation conditions. DUSP2 showed low expression in the peripheral blood serum of AKI patients. In the renal tissues of patients and mouse models of AKI, DUSP2 expression was significantly lower, and HIF‐1α expression was significantly higher. Under hypoxia–reoxygenation conditions, DUSP2 overexpression promoted HK‐2 cell proliferation, inhibited apoptosis and suppressed HIF‐1α expression. The present study demonstrated a significant correlation between DUSP2 and HIF‐1α expression in the context of AKI and revealed the protective effect of DUSP2 overexpression on renal tubular epithelial cells under hypoxia–reoxygenation, which provides a new better understanding of AKI.

## Abstract (highlights)

- **What are the central questions of this study?**
  What is the expression profile of dual‐specificity phosphatase 2 (DUSP2) and hypoxia‐inducible factor‐1α (HIF‐1α) in acute kidney injury and what is the effect of DUSP2 on the function of renal tubular epithelial cells and HIF‐1α expression?
- **What are the main findings and their importance?**
  DUSP2 expression is decreased and HIF‐1α expression is increased in acute kidney injury (AKI). DUSP2 overexpression protects renal tubular epithelial cells under hypoxia–reoxygenation and inhibits HIF‐1α expression in them, which provides a new better understanding of AKI.

## 1 INTRODUCTION

Acute kidney injury (AKI) represents a critical global healthcare challenge. It is defined by rapid deterioration of kidney function and carries significant clinical burdens including elevated in‐hospital mortality rates, heightened long‐term mortality risk, and accelerated progression to chronic kidney disease (CKD) (Guerrero‐Mauvecin et al., 2023). This condition affects up to 50% of critically ill patient populations, frequently manifesting as a multisystem complication through distant organ dysfunction that directly exacerbates its mortality profile (Matsuura et al., 2023). A study published in 2022 based on four large population‐based datasets from Canada, Denmark and the UK showed that the annual incidence of AKI ranged from 134.3 to 162.4 cases per 10,000 person‐years, and the standardized 1‐year mortality rate ranged from 30.4% to 38.5% (Sawhney et al., 2022). Statistically, the incidence of AKI in hospitalized patients reaches 10–15% (Al‐Jaghbeer et al., 2018); in intensive care units, the incidence of AKI is more than 50% (Hoste et al., 2015), and 10.4% of patients are diagnosed with AKI upon arrival at the emergency department (Ehmann et al., 2024). Advances in understanding the underlying pathophysiology of AKI hold the promise of development of new therapeutic strategies to improve the prognosis of patients with AKI (Li et al., 2024). Elucidation of the molecular mechanisms of AKI will provide a pivotal foundation for developing novel therapeutic strategies aimed at improving clinical outcomes in affected patients (Li et al., 2024).

Dual‐specificity phosphatase 2 (DUSP2) is a dual threonine/tyrosine phosphatase that negatively regulates the mitogen‐activated protein kinase (MAPK) family (consisting mainly of the extracellular signal‐regulated kinase (ERK), c‐Jun amino‐terminal kinase, and p38), specifically dephosphorylating and inactivating MAPK (Yin et al., 2003). DUSP2 is predominantly located in the nucleus and nuclear membrane (Mutlak and Kehat, 2021), and plays a crucial role in the regulation of the cell proliferation–apoptosis dynamic balance, inflammatory chain response, and angiogenesis by regulating the nuclear signaling feedback loop of MAPKs (Jiang et al., 2023; Lyu et al., 2024; Mutlak and Kehat, 2021; Shao et al., 2023). DUSP2 demonstrates wide substrate specificity, regulating not only the MAPK pathway but also non‐canonical targets, including signal transducer and activator of transcription (STAT) 3 (Fan et al., 2024), AKT1 (Zhang et al., 2023) and STAT1 (Lu et al., 2015). As a key factor in immune regulation, DUSP2 is intimately linked to immune cell migration, activation and infiltration (including T cells, B cells and macrophages) (Li et al., 2023; Yin et al., 2003). Additionally, DUSP2 expression is significantly reduced in a variety of malignant tumours and plays a role in negatively regulating cancer progression (Zhang et al., 2022). However, there is little literature on the role of DUSP2 in renal pathophysiology. Liu et al. (2023) found that DUSP2 attenuates the inflammatory response and improves renal function in lupus nephritis mice by inhibiting STAT3 phosphorylation. A recent study found that DUSP2 was significantly downregulated in several AKI models, and overexpression of DUSP2 in renal tubular epithelial cells (RTECs) significantly ameliorated AKI (Xiong et al., 2022). The functional mechanism of DUSP2 in AKI and its pathophysiological significance need to be further explored.

Hypoxia‐inducible factor‐1α (HIF‐1α) serves as the master transcriptional regulator orchestrating adaptive cellular to hypoxic stress (Sato and Takeda, 2023). In the kidney, HIF‐1α is expressed by most RTECs (Shu et al., 2019). Under normoxic conditions, HIF‐1α is maintained at low levels in most cells (Wan et al., 2024). However, hypoxic conditions are able to inhibit the degradation of HIF‐1α by the proteasome, allowing HIF‐1α to translocate to the nucleus and dimerize with HIF‐1β, which binds to hypoxia‐responsive elements (HREs) in the promoter regions of target genes, leading to transcriptional activation of hypoxia‐responsive genes (Loboda et al., 2010). As HIF‐1α initiates the upregulation of these genes, they begin to promote angiogenesis, glucose metabolism and cell proliferation, ultimately helping cells to survive in hypoxic environments and thus play a protective role in AKI (Liu, Li et al., 2022; Liu, Zhu et al., 2022; Wei, Xu et al., 2022; Wei, Hou et al., 2022). HIF‐1α mediates metabolic reprogramming in renal cells, resulting in aberrant metabolite accumulation and subsequent activation of pro‐fibrotic signalling pathways (Hu et al., 2024). The expression level and function of HIF‐1α are modulated through multiple post‐translational modifications, while pharmacological interventions targeting HIF‐1α exert therapeutic effects through either suppression of its transcriptional activity or promotion of its proteasomal degradation (Liu et al., 2023).

**FIGURE 1:**
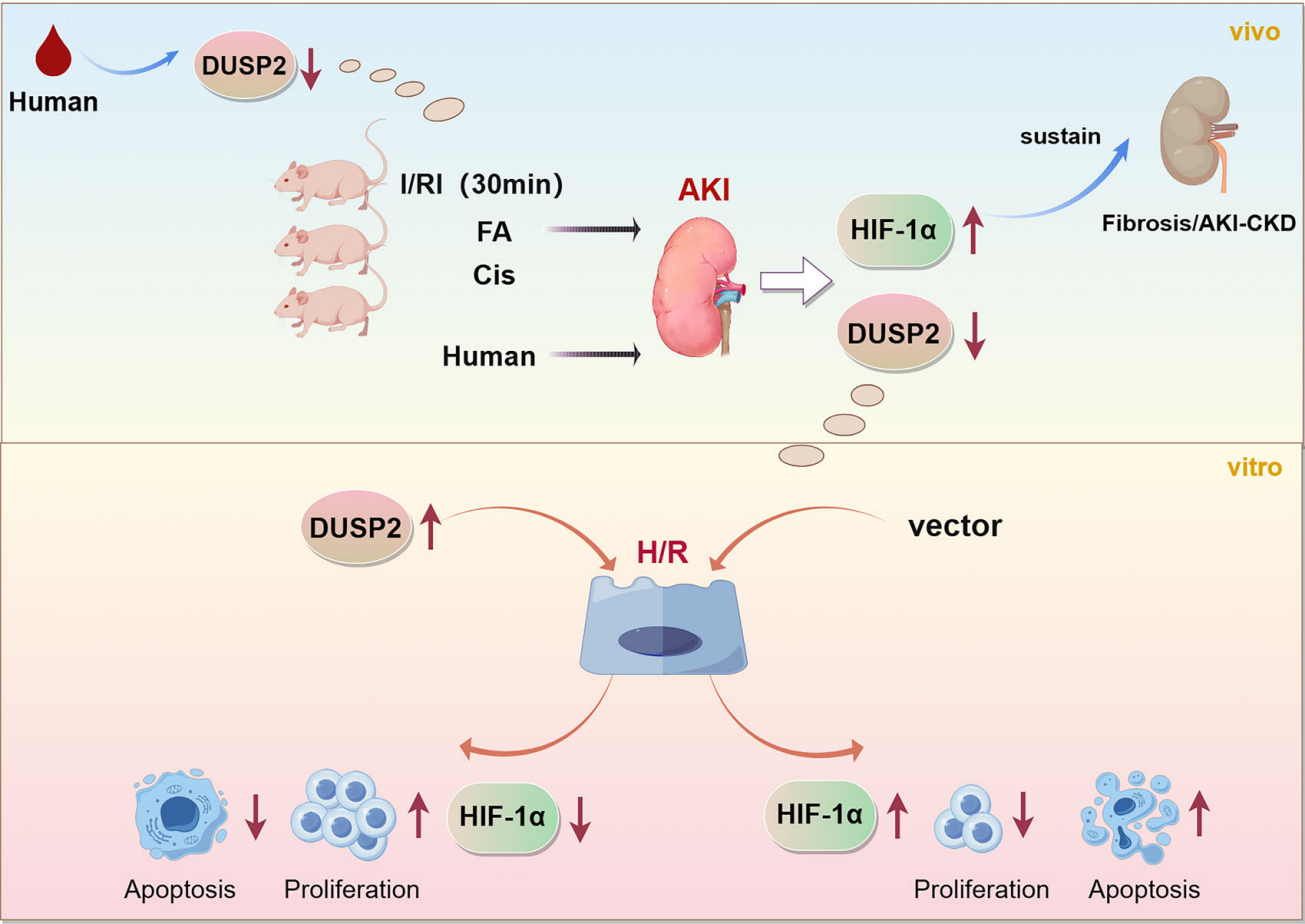
Schematic diagram of the study.In this study, DUSP2 was first found to be expressed at a low level in the peripheral blood of AKI patients. Further investigation revealed that renal tissues of AKI patients and three AKI model mice, namely,Ischemia‐Reperfusion Injury of the Kidney, Folic Acid and Cisplatin, showed elevated expression of HIF‐1α and decreased expression of DUSP2, and that persistent activation of HIF‐1α may lead to renal fibrosis and progression to CKD after AKI. Under hypoxia–reoxygenation induction, human renal cortical proximal tubule epithelial cells exhibited increased apoptosis and decreased cell proliferation as well as elevated HIF‐1α expression during airborne viral infection; in contrast, apoptosis was significantly reduced, cell proliferation was promoted, and HIF‐1α expression was decreased under viral infection with DUSP2 overexpression.

In this study, we used in vitro and in vivo experiments to investigate the characteristic expression changes presented by DUSP2 and HIF‐1α during AKI and verified that overexpression of the DUSP2 state in hypoxia–reoxygenation‐induced human renal tubular epithelial cells (HK‐2) promotes cell proliferation, inhibits apoptosis and suppresses HIF‐1α expression (Fig. 1).

## 2 METHODS

### 2.1 Ethical approval

Written informed consent was obtained from all patients for the required human samples in accordance with the *Declaration of Helsinki*. All animal experimental procedures were conducted in accordance with the guidelines of ethical standards for animal investigations. The project has passed the ethical review and obtained Lun approval documents from the Ethics Committee of the First Affiliated Hospital of Xinjiang Medical University and the Ethical Review Committee of Animal Experimentation of Xinjiang Medical University (Project Approval No. 230306–63).

### 2.2 Human samples

In this study, a total of 60 adult patients attending our hospital from April 2023 to April 2024 were enrolled in a prospective study, of which 30 patients with AKI during hospitalization were selected as the case group, and 30 patients from the population undergoing a physical examination were selected as the control group during the same period. Peripheral blood samples were collected from the patients in the two groups, with 5 mL in each case, for mRNA detection. Subsequently, six pairs of normal tissues adjacent to the lesions of patients who underwent partial nephrectomy in the Urology Center of the First Affiliated Hospital of Xinjiang Medical University were included, and the patients were divided into the disease group and the control group according to the presence of preoperative renal injury.

### 2.3 Animal grouping and experiments

Twenty‐four Specific Pathogen‐Free‐grade 6‐ to 8‐week‐old male C57BL/6 mice weighing 18 ± 2 g were used, purchased from the Animal Centre of Xinjiang Medical University, and housed at a temperature of 23± 3°C, a relative humidity of 40∼70%, 12 hours of light/12 hours of darkness alternate daily cycles, and free access to food and water.

For anaesthesia, 250 mg of Sutent 50 was mixed with 2.5 mL of xylazine hydrochloride (100 mg/mL); 22.5 mL of sterile saline was used to dilute the mixed solution of Sutent 50 (10 mg/mL) and xylazine hydrochloride (10 mg/mL). During intraoperative anaesthesia, the configured solution was injected into the left lower abdomen, avoiding the location of the organs. For diluted cisplatin solution, cisplatin was diluted to 1 mg/mL using 0.9% sterile saline. For diluted folic acid solution: folic acid was diluted to a concentration of 30 mmol/L using 0.3 mol/L sodium bicarbonate. For postoperative analgesic care, buprenorphine 0.05–0.1 mg/kg was injected subcutaneously every 8–12 h.

Twenty‐four mice were randomly divided into four groups: a Sham group, an ischaemia–reperfusion (I/R) kidney injury group (I/R‐AKI), a cisplatin‐induced kidney injury group (Cis‐AKI), and a folic acid‐induced model group (FA‐AKI), with six mice in each group. The control group was randomly divided into two groups of three mice each. One group of mice was anaesthetized with a median longitudinal incision in the abdomen to expose the kidneys – only the renal hilum was exposed, and the hilum was not clamped. The other group of mice was anaesthetized by a single intraperitoneal injection of an equal volume of physiological saline after 72 h. A bilateral I/R injury model was established by clamping the bilateral renal hilum for 30 min using a non‐invasive arterial clip, and then anaesthetizing the mice after reperfusion for 24 h. Mice in the Cis model group were injected with a single intraperitoneal injection of a diluted solution of cisplatin at 20 mg/kg and anaesthetized for 72 h. Mice in the FA model group were injected with a single intraperitoneal injection of a diluted solution of folate at 250 mg/kg and anaesthetized for 48 h. 1 mL of blood was collected from the orbits of the mice in each group, and the levels of BUN and Scr were measured and analysed separately. Sodium pentobarbital (200 mg/kg) was injected intraperitoneally under aseptic conditions to ensure rapid and painless death of the mice, and continuous observation was performed to confirm cardiac and respiratory arrest, dilated pupils, and absence of corneal reflexes. Intact kidney tissue was subsequently removed for analysis.

### 2.4 Histochemical staining

Mouse kidneys were fixed in 4% paraformaldehyde for 24 h, embedded in paraffin, and cut longitudinally into 5 µm‐thick cross‐sections. Sections were stained with trichrome staining kits of haematoxylin and eosin (H&E; ZsBio, Beijing, China), Masson's trichome, and Periodic acid–Schiff (PAS; Solarbio, Beijing, China). The extent of fibrotic areas was measured by Image‐Pro Plus 6.0.

### 2.5 Measurement of Scr and BUN

About 1 mL of blood was taken from mouse orbits using a mixed solution of Sultai 50 (10 mg/mL) + xylazine hydrochloride (10 mg/mL), centrifuged (3000 rpm centrifugal radius: 10 cm, RCF ≈ 1006 ×g) at 4°C for 10 min, and the supernatant was taken in separate fractions and assayed using a Scr kit and a BUN kit (Jiancheng, Nanjing, China).

### 2.6 Cell culture, treatment

HK‐2 cells were obtained from Proximity Bio (Wuhan, China), prepared as a single cell suspension of 1 × 10^5^ cells/mL with complete medium, inoculated into six‐well plates, and cultured in a cell culture incubator at 37°C with saturated humidity and 5% CO_2_. The cells were divided into four groups: blank control group, hypoxia/reoxygenation (H/R) group, H/R+null control group, and H/R+DUSP2 overexpression group. To establish the cell model of H/R, the cells were cultured in 1% O_2_ + 5% CO_2_ + 94% N_2_ for 4 h, then transferred to 20% O_2_ + 5% CO_2_ + 75% N_2_ and reoxygenated for 4, 6, 8 or 12 h, and finally 4 h of hypoxia/6 h of reoxygenation was selected as the test condition.

### 2.7 Construction and transfection of lentiviral vector

The human *DUSP2* gene was integrated into the GV358 lentiviral expression vector, which was purchased from GeneChem (Shanghai, China). The *DUSP2* gene was amplified using PrimeStar HS DNA polymerase (Takara), and the amplified *DUSP2* gene and GV358 vector were digested with a suitable restriction endonuclease (*Age*I) to form compatible ends for ligation. Positive clones were identified by colony PCR and sequenced to confirm the correct insertion of the *DUSP2* gene into the GV358 vector. The GV358‐DUSP2 plasmid, Helper1.0 plasmid, and Helper2.0 plasmid were then co‐transfected into 293T cells. Supernatants from the treated 293T cells were collected and filtered 72 h post‐transfection. HK‐2 cells were transfected with *DUSP2*‐overexpressing lentivirus using viral co‐infection reagent HiTransG P, multiplicity of infection (MOI) = 100, for 72 h for subsequent experiments.

### 2.8 CCK8 assay for cell proliferation

HK‐2 cells (5 × 10^4^ cells/well) were inoculated into 96‐well culture plates and incubated overnight; 100 µL of configured 10% CCK‐8 solution (All Style Gold Bio, Beijing, China) was added to each well at 37°C and incubated for another 1 h. Absorbance was measured at 450 nm using an enzyme meter.

### 2.9 TUNEL detection of apoptosis

HK‐2 apoptosis was detected using a terminal deoxynucleotidyl transferase dUTP nick end labelling (TUNEL) kit (BOSTER, Wuhan, China). The slides with adherent cells were fixed with 4% paraformaldehyde for 30 min at room temperature. After washing with phosphate‐buffered saline (PBS), 20 µL of TUNEL reaction mix was incubated with the sections for 2 h. The sections were further washed with PBS and incubated with 4′,6‐diamidino‐2‐phenylindole (DAPI) solution for 5 min in the dark. Apoptosis was observed under a fluorescence microscope. TUNEL‐positive cells were quantified.

### 2.10 qRT‐PCR assay

Total RNA from the cells was extracted using a TRIzol reagent; 1000 ng of total RNA was reverse transcribed to cDNA using 5X All‐In‐One RT MasterMix (G492, ABM, Richmond, Canada) for each sample; 1000 ng of total RNA was reverse transcribed to cDNA using BlasTaq 2× qPCR MasterMix and a MyCycler Thermal Cycler real‐time PCR machine (Bio‐Rad Laboratories, Hercules, CA, USA) to measure mRNA levels, and the relative expression of mRNA was determined by the $2^{-\Delta \Delta C_{t}}$ method. The relative expression level of each gene was normalized to the *β‐Actin* gene. The primer sequences were as follows: *DUSP2*‐F1: 5′‐TGTGGAGATCTTGCCCTACC‐3′, *DUSP2*‐R1: 5′‐AAAAGGCCCTCAAAGTGGTT‐3′, *HIF1α*‐F1: 5′‐GCCGCTGGAGACACAATCATA‐3′, *HIF1α*‐R1: 5′‐GGTGAGGGGAGCATTACATCAT‐3′, *GAPDH*‐F1: 5′‐TGTTGCCATCAATGACCCCTT‐3′, *GAPDH*‐R1: 5′‐CTCCACGACGTACTCAGCG‐3′.

### 2.11 Western blot assay

Proteins were lysed with RIPA buffer, to which protease inhibitors and phosphatase inhibitors were added. Protein samples extracted from renal cortex and HK‐2 cells were loaded onto SDS‐PAGE gels (Sangon Biotech, Shanghai, China) and transferred onto polyvinylidene difluoride (PVDF) membranes (Millipore, Billerica, MA, USA). The PVDF membrane was incubated with the primary antibody at 4°C overnight. The membranes were washed and incubated with anti‐rabbit or anti‐mouse (1:10 000) secondary antibodies for 50 min at room temperature without light. Primary antibodies used were β‐actin (1:1000), DUSP2 (1:500, 27327‐1‐AP, Triple Eagle, Wuhan, China) and HIF‐1α (1:600, ab179483, Abcam, Waltham, MA, USA), and secondary antibodies used were goat anti‐mouse IgG Heavy Chain & Light Chain（H&L ）(horseradish peroxidase (HRP)) (1:10000, ab205719, Abcam) and goat anti‐rabbit IgG H&L (HRP) (1:5000, ab205718, Abcam). The membranes were detected and photographed using a ChemiScope mini chemiluminescence instrument from Shanghai (China) Qinxiang Scientific Instrument Co.

### 2.12 Data statistics

All results are expressed as means ± standard deviation. Statistical analysis was performed using GraphPad Prism 9.5.0 (GraphPad Software, San Diego, CA, USA). Student's *t*‐test was used to test the difference between the two groups, one‐way ANOVA was used for comparison between multiple groups, and Tukey's multiple comparison test was used for multiple comparisons. A *P*‐value < 0.05 was considered statistically significant.

## 3 RESULTS

### 3.1 At the mRNA level, DUSP2 expression was downregulated in peripheral blood and renal tissues, and HIF‐1α expression was upregulated in renal tissues of AKI patients

**FIGURE 2:**
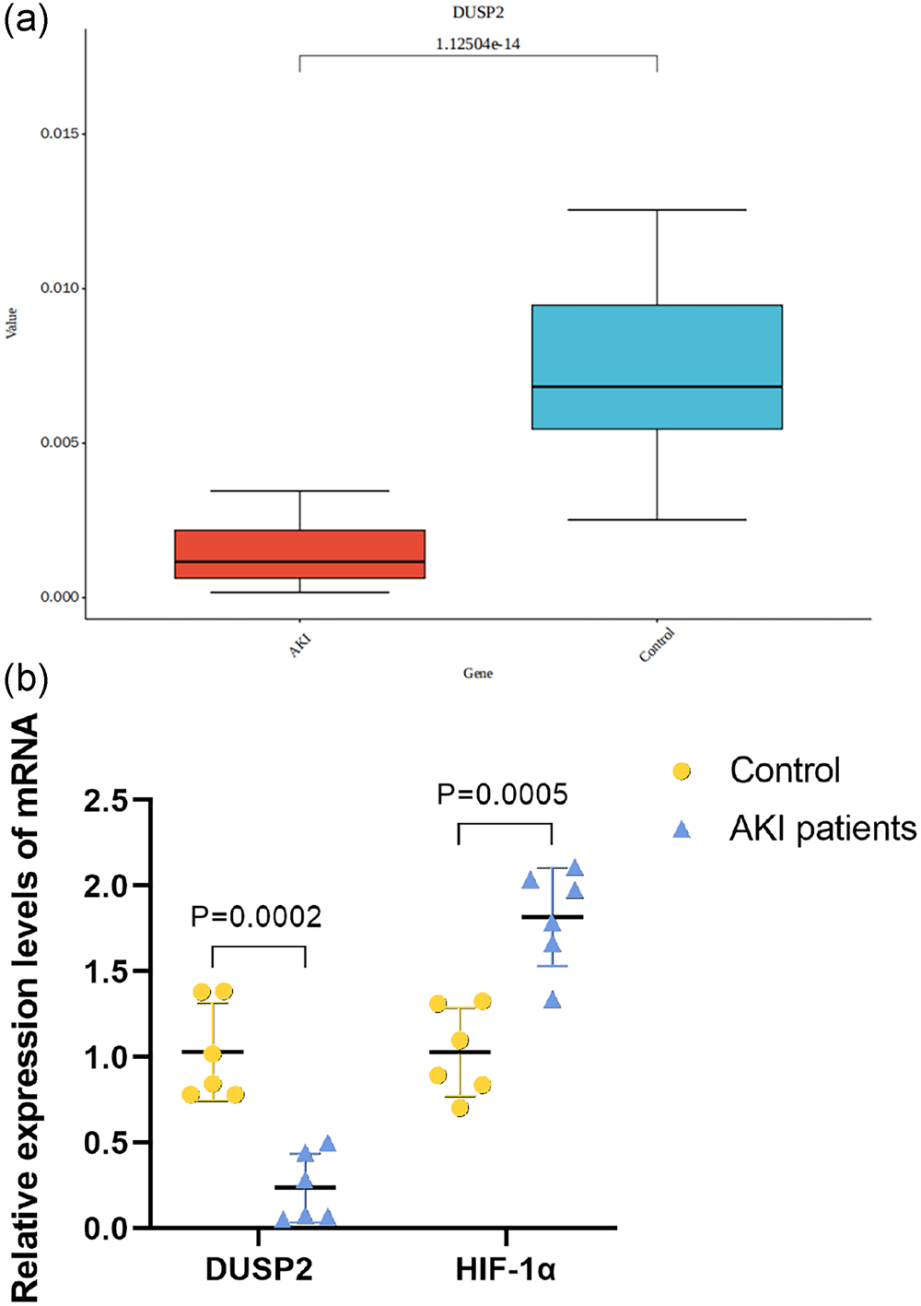
DUSP2 expression was downregulated in peripheral blood and renal tissues of AKI patients, and HIF‐1α expression was upregulated in renal tissues of AKI patients. (a) DUSP2 levels in peripheral blood of ischaemia–reperfusion AKI patients and non‐AKI patients. Data are expressed as means ± SD; *n* = 30, *P *< 0.05. (b) qRT‐PCR assay to analyse the changes of mRNA expression of DUSP2 and HIF‐1α genes in renal tissues of control and AKI patients (data are expressed as means ± SD, *n* = 6. *P *< 0.001). AKI, acute kidney injury; DUSP2, dual‐specificity phosphatase 2; HIF‐1α, hypoxia‐inducible factor‐1α. *P* < 0.05: Indicates a statistically significant difference, meaning there is less than a 5% probability that the observed difference is due to random chance.*P* < 0.001: Indicates an extremely statistically significant difference, where the probability of the result being caused by random chance is less than 0.1%.

DUSP2 levels in the peripheral blood serum of patients in the case and control groups were first determined (*n* = 30). The results showed that DUSP2 was differentially expressed in the AKI and non‐AKI groups, and DUSP2 in the serum of the AKI group showed significantly lower expression (Figure 2a).

Using qRT‐PCR to detect the mRNA expression levels of DUSP2 and HIF‐1α genes in the kidney tissues of patients in the disease and control groups, it was found that the mRNA expression levels in the two groups were also differentially expressed, and the mRNA level of DUSP2 gene in the kidney tissues of AKI patients was significantly lower than that of non‐AKI patients, while the mRNA expression of HIF‐1α gene was significantly higher compared to non‐AKI patients (Figure 2b).

### 3.2 Downregulation of DUSP2 expression and upregulation of HIF‐1α expression in kidney tissues of mice with different AKI types

**FIGURE 3:**
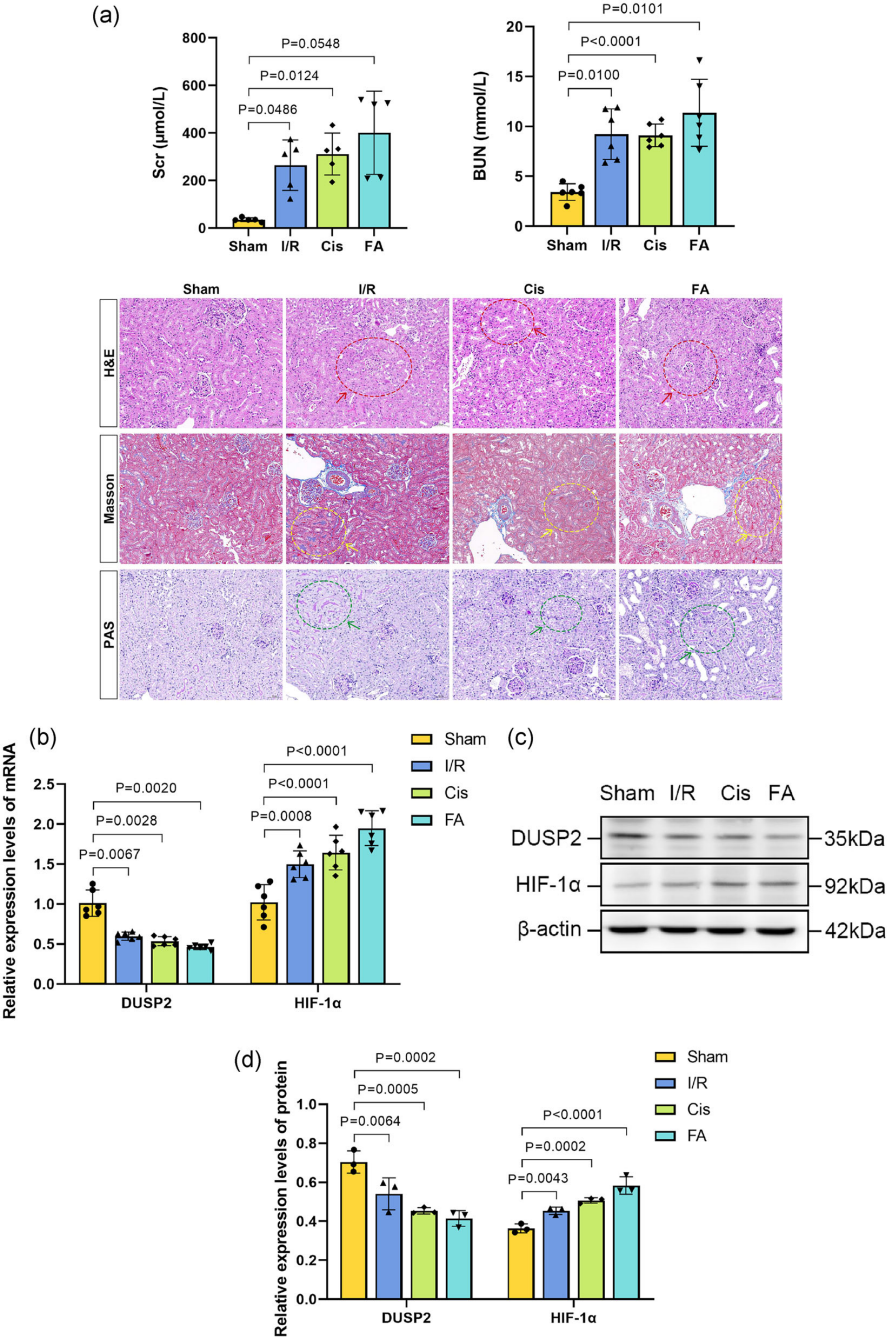
Downregulation of DUSP2 expression and upregulation of HIF‐1α expression in kidney tissues of mice with different AKI type. (a) A kit was used to analyse the changes of Scr and BUN in the serum of different types of AKI mice. The data are expressed as means ± SD; *n* = 5 or 6, *P *< 0.05, *P *< 0.01. Haematoxylin and eosin (H&E) staining was used to observe the pathological damage of renal tissues of different types of AKI mice. Masson staining was used to observe the degree of collagen fibrosis in the kidney tissues of different types of AKI mice. PAS staining was used to observe the glycogen deposition in kidney tissues of different types of AKI mice. Magnification, ×400, Scale bars, 20 µm, *n* = 6. (b) qRT‐PCR was performed to detect the changes in mRNA expression of DUSP2 and HIF‐1α genes in renal tissues of different types of AKI mice, and the data are expressed as means ± SD; *n* = 3, *P *< 0.01, *P *< 0.001. (c) Western blot assay performed to analyse the changes in mRNA expression of the different types of representative pictures of DUSP2 and HIF‐1α protein bands in kidney tissues of AKI mice. (d) qRT‐PCR assay to analyse the expression changes of DUSP2 and HIF‐1α proteins in kidney tissues of different types of AKI mice. The data are expressed as means ± SD; *n* = 3. *P *< 0.01, *P *< 0.001. AKI, acute kidney injury; DUSP2, dual‐specificity phosphatase 2; HIF‐1α, hypoxia‐inducible factor‐1α. *P* < 0.05: Indicates a statistically significant difference, meaning there is less than a 5% probability that the observed difference is due to random chance. *P* < 0.01: Indicates a highly statistically significant difference, with less than a 1% probability of the difference arising from random variability. *P* < 0.001: Indicates an extremely statistically significant difference, where the probability of the result being caused by random chance is less than 0.1%.

Using the kit to detect the levels of Scr and BUN in the serum of mice in each group, the results showed that Scr and BUN in the serum of the three groups of AKI model mice were significantly higher than those in the sham operation group (Sham group). We observed the renal tissues of mice in each group by haematoxylin and eosin staining, and found that in the Sham group the renal tissues were basically structurally intact, the glomerular tissues were well‐structured and normal in number, the brush border of renal tubular epithelial cells was well defined, and the nuclei and basement membranes were intact; the renal tissues of the three groups of AKI model mice showed varying degrees of renal tubular epithelial cell detachment necrosis, tubular lumen enlargement, and some interstitial oedema, focal lysis and necrosis. In the renal tissues of mice with the SI model, different degrees of tubular epithelial cell detachment and necrosis were seen. It was observed by Masson staining that renal interstitial fibrous tissue proliferation was not obvious and the proportion of fibrosis was low in the Sham group; renal interstitial fibrous tissue proliferation was mildly increased and the proportion of fibrosis was increased in the three groups of AKI model mice. It was observed by PAS staining that the renal tissues of the Sham group were largely normal in structure, and the purplish‐red glycogen deposition in the renal tubular epithelium was not obvious; the renal tubules of the three groups of AKI model mice were more disorganized in structure, and the purplish‐red glycogen deposition was more obvious. The results of serology and mouse pathology (Figure 3a) indicated that we successfully constructed the AKI mouse model.

Changes in mRNA and protein expression of DUSP2 and HIF‐1α genes in kidney tissues of different types of AKI mice were detected using qRT‐PCR. A western blot assay was used to analyse DUSP2 and HIF‐1α proteins in kidney tissues of different types of AKI mice. These results (Figure 3b–d) showed that AKI induced by different causes resulted in downregulation of DUSP2 expression and upregulation of HIF‐1α expression in renal tissues compared with non‐AKI controls.

### 3.3 H/R‐induced downregulation of DUSP2 expression in HK‐2 cells

**FIGURE 4:**
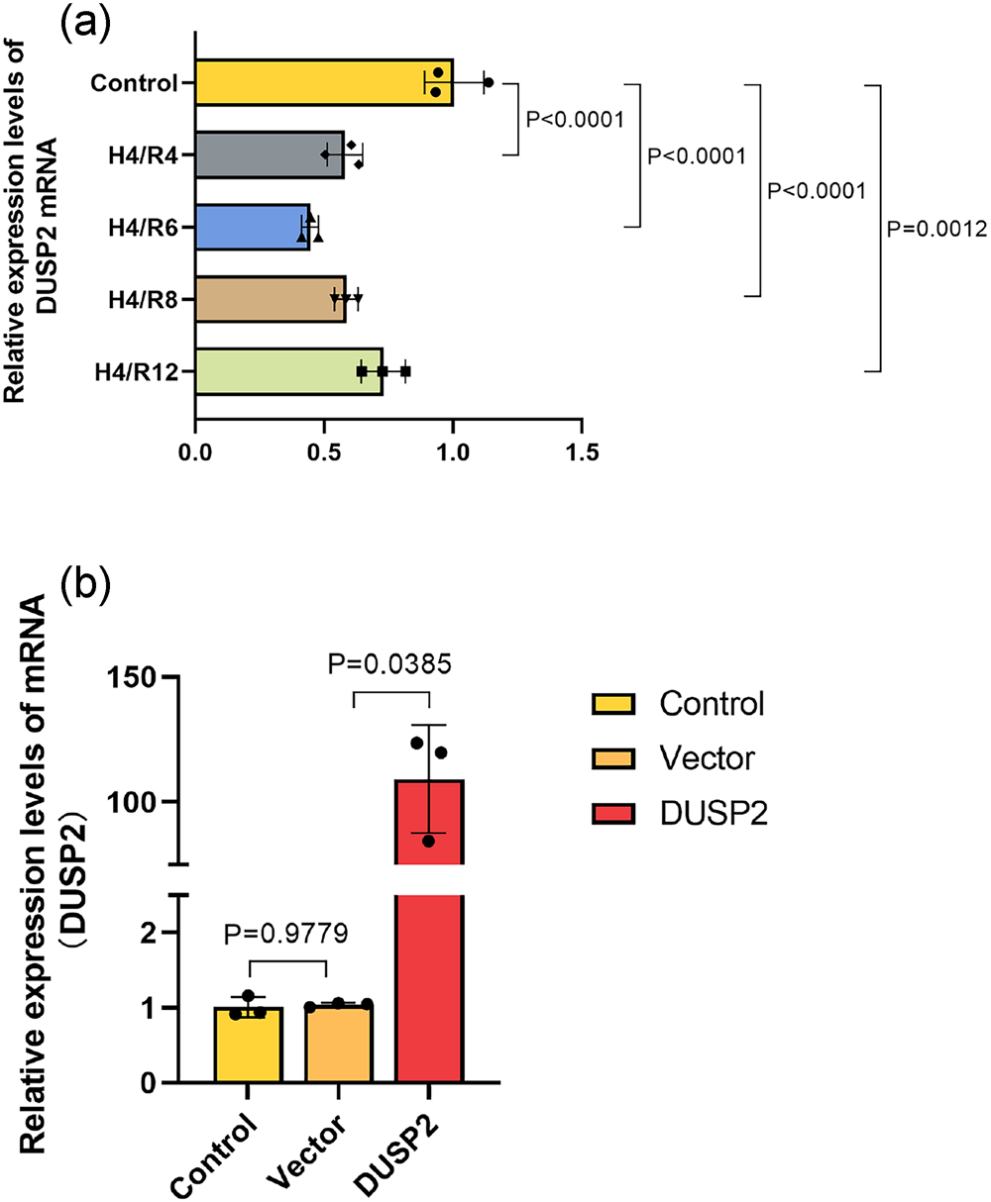
H/R‐induced downregulation of DUSP2 expression in HK‐2 cells. (a) A qRT‐PCR assay was performed to analyse the changes in mRNA expression of the DUSP2 gene in HK‐2 cells in different H/R induction conditions. Data are expressed as means ± SD; *n* = 3, *P *< 0.01, *P *< 0.001. (b) A qRT‐PCR assay was performed to detect the expression of mRNA to verify the overexpression efficiency of the DUSP2 gene in HK‐2 cells. Data are expressed as mean ± SD, *n* = 3; ns, *P *> 0.05, *P *< 0.05. AKI, acute kidney injury; DUSP2, dual‐specificity phosphatase 2; HIF‐1α, hypoxia‐inducible factor‐1α; H/R, hypoxia/reoxygenation. *P* < 0.05: Indicates a statistically significant difference, meaning there is less than a 5% probability that the observed difference is due to random chance. *P* < 0.01: Indicates a highly statistically significant difference, with less than a 1% probability of the difference arising from random variability. *P* < 0.001: Indicates an extremely statistically significant difference, where the probability of the result being caused by random chance is less than 0.1%.

Next, an in vitro H/R model was established using HK‐2 cells, and the mRNA levels of DUSP2 in HK‐2 cells were analysed under different H/R conditions. The results (Figure 4a) showed that, consistent with the in vivo results, DUSP2 expression in HK‐2 cells under different H/R‐induced conditions was lower than that in the blank control group. Hypoxia 4 h/reoxygenation 6 h was the optimal induction condition, and DUSP2 expression was lowest in HK‐2 cells under this H/R condition.

The overexpression efficiency of the DUSP2 gene in HK‐2 cells was verified by detection of mRNA expression by qRT‐PCR (Figure 4b).

### 3.4 DUSP2 overexpression promotes proliferation and inhibits apoptosis in H/R‐induced HK‐2

**FIGURE 5:**
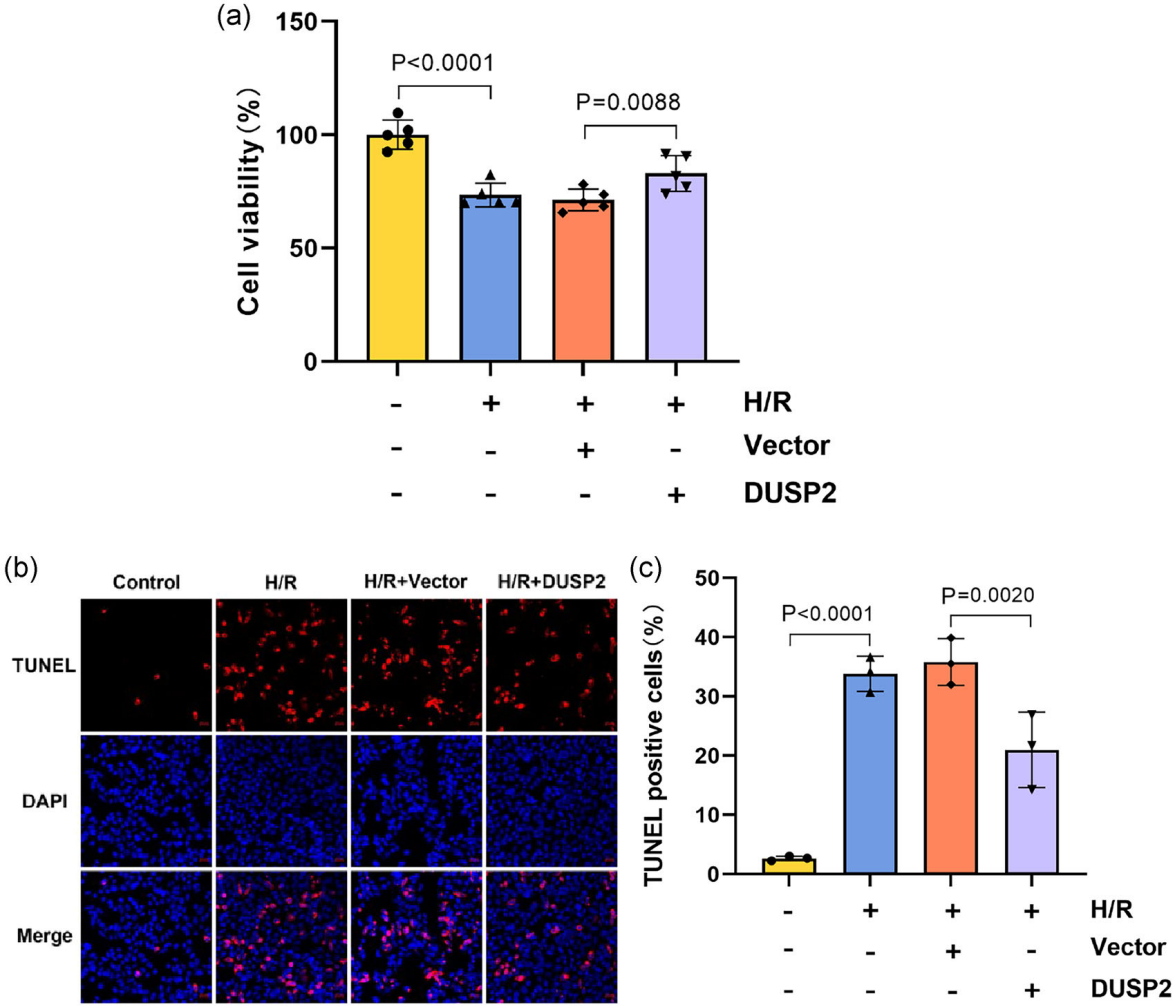
DUSP2 overexpression plays a role in promoting proliferation and inhibiting apoptosis in H/R‐induced HK‐2. (a) CCK8 assay was used to analyse the changes in proliferative viability of H/R‐induced HK‐2 cells, and the data are expressed as means ± SD; *n* = 5, *P *< 0.001. (b) Representative images of H/R‐induced apoptosis in hypoxia‐induced HK‐2 cells by immunofluorescence staining. CY3 fluorescent probe (red) labelling was used to represent TUNEL‐stained positive cells, and DAPI dye (blue) labelling was used to represent nuclei. Scale bars, 20um, *n* = 3. (c) Quantitative immunofluorescence analysis of H/R‐induced apoptosis of HK‐2 cells. Data are expressed as means ± SD; *n* = 3, *P *< 0.01, *P *< 0.001. AKI, acute kidney injury; DUSP2, dual‐specificity phosphatase 2; HIF‐1α, hypoxia‐inducible factor‐1α; H/R, hypoxia/reoxygenation.

A CCK‐8 assay showed (Figure 5a) that the proliferative activity of HK‐2 cells in the H/R group and the H/R+null control group was significantly lower than that in the blank control group (*P *< 0.001), while the mRNA expression level of HIF‐1α gene in the H/R+DUSP2 overexpression group was significantly higher than that in the H/R group and the H/R+null control group. These findings suggest that DUSP2 overexpression partially restored the cell proliferation inhibited by HK‐2 under H/R induction.

Observation and quantitative analysis using double immunofluorescence staining revealed that HK‐2 apoptosis was higher in the H/R+DUSP2 overexpression group, H/R group and H/R+null control group than in the blank control group (*P *< 0.001), but was lower in the H/R+DUSP2 overexpression group than in the H/R group and the H/R+null control group (*P *< 0.01) (Figure 5b,c). These results indicated that apoptosis of HK‐2 was increased under H/R induction, but DUSP2 overexpression was able to significantly inhibit the apoptotic situation under this condition.

### 3.5 DUSP2 overexpression inhibits H/R‐induced HIF‐1α gene expression levels

**FIGURE 6:**
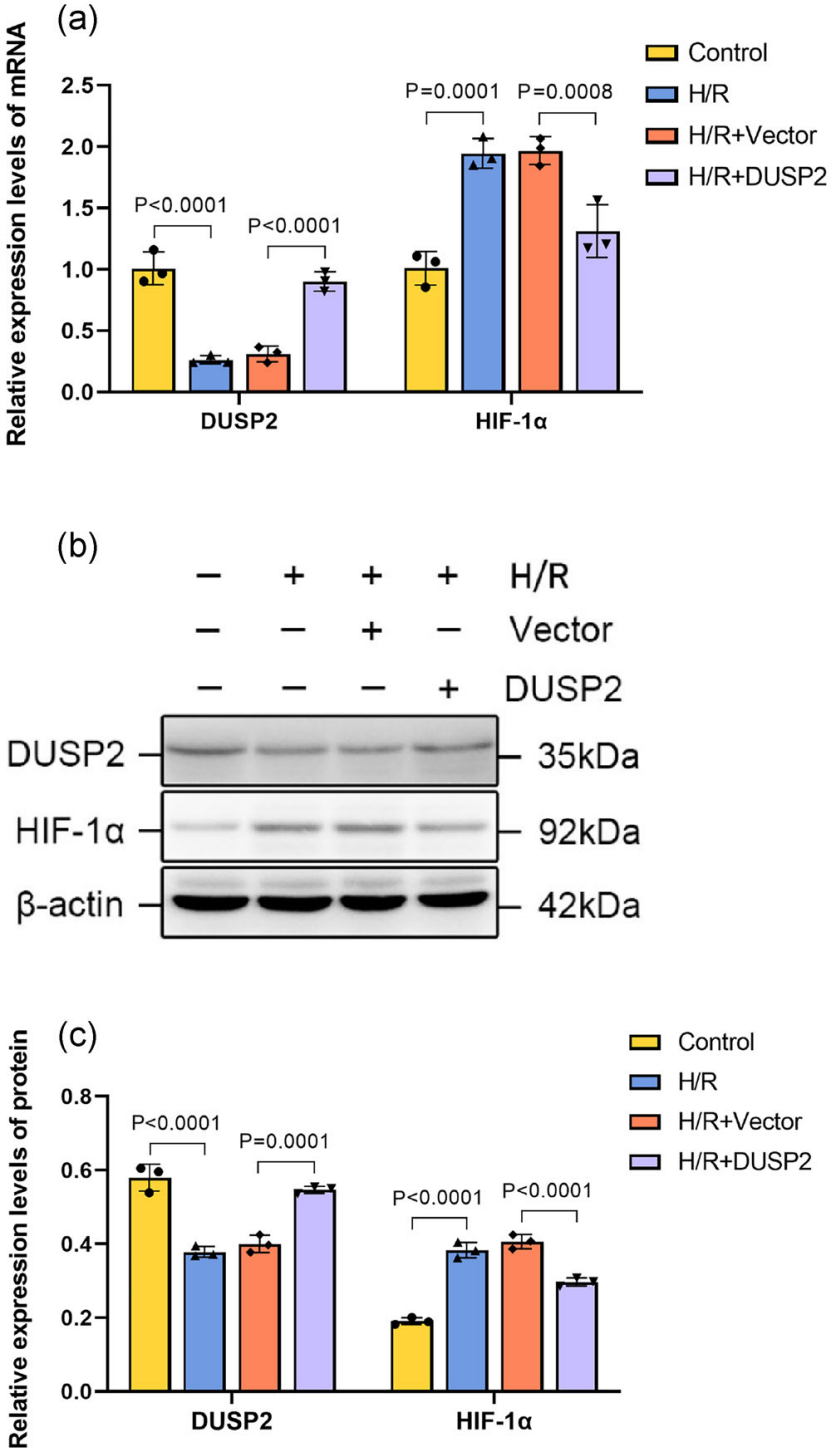
DUSP2 overexpression inhibited H/R‐induced HIF‐1α gene expression level. (a) qRT‐PCR assay was performed to analyse the changes of DUSP2 and HIF‐1α gene mRNA expression in H/R‐induced HK‐2 cells. Data are expressed as means ± SD; *n* = 3, *P *< 0.001. (b) Representative images of DUSP2 and HIF‐1α protein bands in H/R‐induced HK‐2 cells analysed by western blot assay. (c) Western blot densitometric quantification of H/R‐induced changes in the expression of DUSP2 and HIF‐1α proteins in HK‐2 cells. Data are expressed as means ± SD; *n* = 3, *P *< 0.001. AKI, acute kidney injury; DUSP2, dual‐specificity phosphatase 2; HIF‐1α, hypoxia‐inducible factor‐1α; H/R, hypoxia/reoxygenation.

The mRNA expression levels of DUSP2 and HIF‐1α genes in H/R‐induced HK‐2 were detected using qRT‐PCR, which showed that the mRNA expression levels of HIF‐1α genes in the H/R group and the H/R+null control group were significantly higher than those in the blank control group (*P *< 0.001), whereas the mRNA expression of HIF‐1α genes in the H/R+DUSP2 overexpression group was significantly lower than that in the H/R group and the H/R+ blank control group (Figure 6a).

DUSP2 and HIF‐1α proteins in HK‐2 under H/R conditions were detected using western blot, and representative images of protein bands are shown in Figure 6b. Then, the changes of DUSP2 and HIF‐1α protein expression in H/R‐induced HK‐2 were quantitatively analysed using western blot densitometry, and the results (Figure 6b) showed that the expression levels of HIF‐1α protein were significantly higher in the H/R group and the H/R+null control group than in the blank control group (*P *< 0.001), whereas the expression levels of HIF‐1α protein were significantly lower in the H/R+DUSP2 overexpression group than in the H/R group and the H/R+null control group. Thus, the mRNA and protein expression of HIF‐1α in HK‐2 was increased under H/R induction, which was significantly suppressed when DUSP2 was overexpressed, resulting in a decrease in the magnitude of the H/R‐induced increase in HIF‐1α expression, which suggests that DUSP2 suppressed the expression of HIF‐1α under H/R.

## 4 DISCUSSION

AKI is a common clinical critical condition with diverse aetiologies and complex mechanisms, which not only affects short‐term health but also increases the risk of CDK, cardiovascular complications and death (Ehmann et al., 2024). Dual‐specificity phosphatases (DUSPs) function as critical regulatory nodes in renal pathophysiology, and the expression levels of most species of DUSPs are decreased in renal disease models, whereas targeting restoration of DUSP expression attenuates disease‐relevant phenotypic manifestations (Li et al., 2022). DUSP2 is critical in inflammation and immunity (Li et al., 2023; Lyu et al., 2024), yet its role in AKI has rarely been explored. Xiong et al. found that overexpression of DUSP2 significantly ameliorated AKI‐induced renal tubular injury and interstitial inflammation by inactivating STAT1 to limit gasdermin D‐mediated cellular pyroptosis in RTEC cells (Xiong et al., 2022). Thus, in the present study, we first examined DUSP2 levels in the peripheral blood serum of AKI patients and non‐AKI patients and clarified that DUSP2 was had low expression in the peripheral blood of AKI patients.

DUSP2 is able to interact directly with the ERK family, impairing ERK activity through its phosphatase function (Jeffrey et al., 2006; Li et al., 2023). In contrast, ERK1/2 regulates the interaction of HIF‐1α with the major export protein CRM1 and the histone molecular chaperone NPM1 by phosphorylating the C‐terminus of HIF‐1α, which promotes the accumulation and transcriptional activation of HIF‐1α in the nucleus (Koukoulas et al., 2021). Elevated HIF‐1α has been demonstrated to be a hallmark change of ischaemia–reperfusion injury (IRI)‐AKI (Zhang et al., 2016). Li et al. found that AKI‐mediated macrophage‐dependent inflammation enhanced HIF‐1α transcription by prompting nuclear factor κB (NF‐κB) to bind to the promoter of HIF‐1α. (Li et al., 2021). Some scholars have constructed different types of AKI models and found that the levels of HIF‐1α in renal tubular cells were significantly increased in AKI caused by I/R, unilateral ureteral obstruction, or sepsis (Liu et al., 2021). In this study, we confirmed that the renal tissues of AKI patients and three typical mouse models of AKI showed a characteristic expression difference of significantly lower DUSP2 expression and significantly higher HIF‐1α expression, suggesting a correlation between the two expressions.

Renal IRI is a common cause of AKI, which may occur in clinical settings such as cardiac surgery, sepsis and shock (Pabla and Bajwa, 2022). IRI is a pathological state in which organs experience a temporary restriction of the blood supply, leading to insufficient oxygen supply and accumulation of metabolic waste products, and the subsequent restoration of perfusion and oxygenation tends to exacerbate the tissue damage and trigger a severe inflammatory response (Eltzschig and Eckle, 2011; Fan et al., 2023). The results of the present study showed that the expression of DUSP2 was significantly downregulated in HK‐2 cells under different H/R conditions. The pathophysiology of IRI is complex, characterized by renal tubular injury, oxidative stress and inflammation, with apoptosis of renal tubular cells being the central mechanism that triggers AKI (Fan et al., 2023;Kanagasundaram, 2015). Recovery of tubular integrity after AKI is mainly dependent on the effective proliferation of RTECs (Alcalay and Vanden Heuvel, 2009). DUSP2 is involved in the regulation of cell proliferation and apoptosis through multiple pathways, and its function varies when bound to different substrates (Zhang et al., 2023). For example, DUSP2 downregulates ERK/MAPK kinase (MEK) and STAT3 signalling pathways in bladder cancer cells and uterine ectopic stromal cells, respectively, which inhibits cell proliferation and promotes apoptosis (Hsiao et al., 2017; Zou et al., 2023), and it activates the MAPK/mechanistic target of rapamycin (mTOR) pathway to promote the proliferation of prostate cancer cells (Ren et al., 2024). CCK‐8 and immunofluorescence staining showed that DUSP2 overexpression was capable of partially restoring H/R‐induced proliferation inhibition and attenuating apoptosis in HK‐2 cells, revealing that DUSP2 plays a protective role in IRI‐AKI. Notably, HIF‐1α was observed to inhibit DUSP2 expression under hypoxic conditions mediated by various cancers, and downregulation of DUSP2 resulted in prolonged ERK phosphorylation, which in turn further increased the cytosolic accumulation and transcriptional levels of HIF‐1α (Karakashev and Reginato, 2015;Lin et al., 2011). In this study, we found that DUSP2 overexpression suppressed HIF‐1α expression in HK‐2 cells under hypoxia–reoxygenation conditions, suggesting a potential regulatory relationship between DUSP2 and HIF‐1α. Combined with previous reports (Lin et al., 2011), this regulatory mechanism may be related to the regulation of ERK phosphorylation by DUSP2, which requires further investigation in future studies.

Short‐term expression of HIF‐1α is beneficial, but sustained activation may lead to chronic inflammation and fibrosis in the kidney (Rahbar et al., 2023; Wei, Xu et al., 2022; Xu et al., 2021). Han et al. found that knockdown of the S100A16 gene in rat RTECs reduced IRI‐induced HIF‐1α expression, which inhibited H/R‐induced apoptosis and transforming growth factor β1‐induced transcription of renal fibrosis factor (Han et al., 2024). It was shown that, unlike the transient expression of HIF‐1α in a moderate injury model with 30 min of I/R treatment, HIF‐1α was persistently activated up to day 14 with renal fibrosis expression in a severe kidney injury model with 40 min of I/R treatment, suggesting that the progression of AKI to CKD is associated with the persistent activation of HIF‐1α. They subsequently demonstrated that activation of the HIF‐1α C‐terminus induces *KLF5* transcription, which promotes renal progression to CKD after severe AKI, and inhibition of its activation reverses this process (Li, Xiong et al., 2021; Li, Wang et al., 2021). In addition, activation of HIF‐1α in mild IRI enhances nuclear factor E2‐related factor 2 (NRF2) activity and promotes repair, and elevated HIF‐1α activity in severe IRI inhibits the nuclear localization and activity of NRF2, mediating the adverse renal regression toward fibrosis and CKD (Bondi et al., 2022). The H/R model in the present study used hypoxia for 4 h as the induction condition, which achieved the condition of constructing severe AKI and inducing sustained activation of HIF‐1α in the above study; therefore, overexpression of DUSP2 in the present study may also have prevented the transition from AKI to CKD by decreasing the expression level of HIF‐1α.

In summary, we verified that DUSP2 expression was significantly reduced and HIF‐1α expression was significantly increased in renal tissues of patients and mouse models of AKI, both of which showed this specific relationship, and that under ischaemic and hypoxic conditions, DUSP2 overexpression could promote HK‐2 cell proliferation, inhibit apoptosis, and inhibit the expression of HIF‐1α, which provided new thoughts. The specific regulatory mechanisms of DUSP2 and HIF‐1α have not yet been clarified, and further studies are needed to better understand their precise roles and potential therapeutic applications.

## AUTHOR CONTRIBUTIONS

The experiments were performed in the State Key Laboratory of Pathogenesis, Prevention and Treatment of High Incidence Diseases in Central Asia, Xinjiang, China. *Experimental data*: Lanyan Lin and Zulibiya·Tuohetiyaer. *Visualization*: Wulaer·Adeli and Sisi Chen. *Writing, original draft preparation*: Songmin Xue and Xueqian Chu. *Review and editing*: Xuan Huang, Suhua Li, and Chen Lu. *Funding acquisition*: Suhua Li. The authors critically reviewed the content of the article. All authors reviewed the final version of the manuscript and agreed to take responsibility for each part of the article and to ensure that any questions of accuracy or completeness of any part of the article were properly verified and addressed. All persons designated as authors are eligible for authorship, and all persons eligible for authorship are listed.

## CONFLICT OF INTEREST

The authors declare that there is no conflict of interest regarding the publication of this paper.

## Data Availability

All authors declared that all data from this study are available.
